# Quantifying cell cycle regulation by tissue crowding

**DOI:** 10.1016/j.bpj.2024.05.003

**Published:** 2024-05-07

**Authors:** Carles Falcó, Daniel J. Cohen, José A. Carrillo, Ruth E. Baker

**Affiliations:** 1 Mathematical Institute, University of Oxford, Oxford, United Kingdom; 2 Department of Mechanical and Aerospace Engineering, Princeton University, Princeton, New Jersey; 3 Department of Chemical and Biological Engineering, Princeton University, Princeton, New Jersey

## Abstract

The spatiotemporal coordination and regulation of cell proliferation is fundamental in many aspects of development and tissue maintenance. Cells have the ability to adapt their division rates in response to mechanical constraints, yet we do not fully understand how cell proliferation regulation impacts cell migration phenomena. Here, we present a minimal continuum model of cell migration with cell cycle dynamics, which includes density-dependent effects and hence can account for cell proliferation regulation. By combining minimal mathematical modeling, Bayesian inference, and recent experimental data, we quantify the impact of tissue crowding across different cell cycle stages in epithelial tissue expansion experiments. Our model suggests that cells sense local density and adapt cell cycle progression in response, during G1 and the combined S/G2/M phases, providing an explicit relationship between each cell-cycle-stage duration and local tissue density, which is consistent with several experimental observations. Finally, we compare our mathematical model’s predictions to different experiments studying cell cycle regulation and present a quantitative analysis on the impact of density-dependent regulation on cell migration patterns. Our work presents a systematic approach for investigating and analyzing cell cycle data, providing mechanistic insights into how individual cells regulate proliferation, based on population-based experimental measurements.

## Significance

The correct regulation of cell proliferation is crucial for the emergence of collective cell behavior during tissue morphogenesis, homeostasis, and regeneration. Moreover, uncontrolled cell division often leads to tumor formation. Here, we propose a mathematical model of cell migration with cell cycle dynamics that accounts for density-dependent effects regulating cell cycle progression. Our model is capable of describing the spatiotemporal cell cycle dynamics observed during epithelial tissue expansion. By combining experimental data, Bayesian inference, and minimal modeling, we describe how each cell cycle phase depends on local cell density, and we quantify the impact of tissue crowding on cell proliferation patterns.

## Introduction

The coordination of cell proliferation across space and time is crucial for the emergence of collective cell migration, which plays a fundamental role in development, including tissue formation and morphogenesis, and also at later stages for tissue regeneration and homeostasis. Cells adapt their division rates in response to mechanical constraints within tissues (1,2), allowing cell populations to self-organize and eventually form and maintain tissues and complex structures. Moreover, disruptions in the control of cell proliferation often result in tumor formation (3,4,5). Although significant experimental efforts have been devoted to understand the mechanical regulation of cell proliferation (6) and its interplay with collective cell migration, existing mathematical models have failed to describe these constraints and how they affect cell cycle progression (7,8,9).

**Figure 1:**
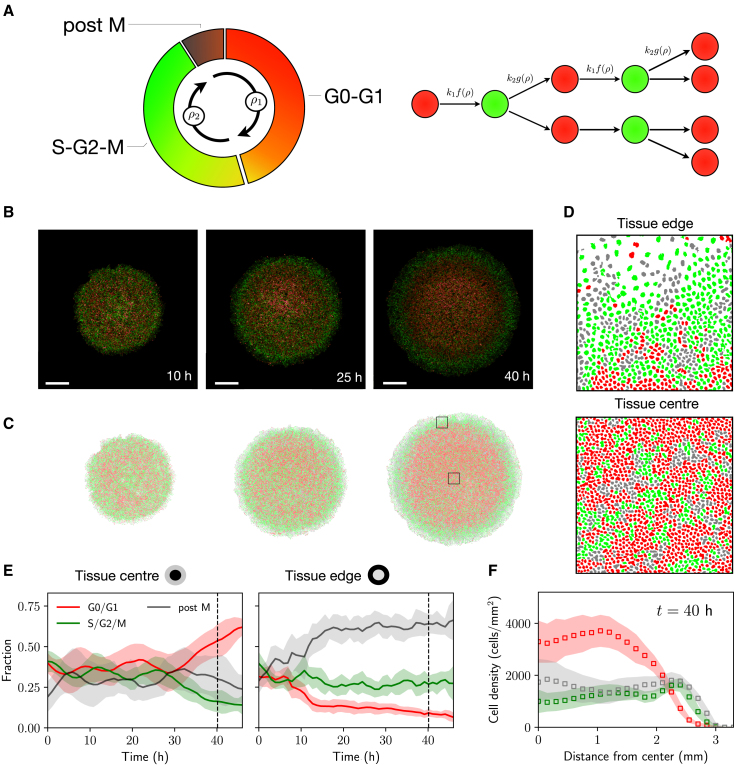
Spatiotemporal dynamics of cell cycle. (*A*) Schematics of the FUCCI cell cycle marker system and model conceptualization. Transitions in the model given by Eq. (1) are regulated by the crowding functions f(ρ) and g(ρ), dependent on the total cell density $\rho =\rho_{1}+\rho_{2}$. (*B*) FUCCI fluorescence images from the experiments of Heinrich et al. (19) at different time points (adapted). Initial tissue diameter ∼3.4 mm. Scale bars correspond to 1 mm. (*C*) Segmented data showing G1 (*red*), S/G2/M (*green*), and postmitotic (*gray*) cells. Note that in the model we combine postmitotic cells and cells in G1. (*D*) Zoomed-in segmented data at the tissue edge and center, corresponding to the black squares in (*C*). (*E*) Fraction of cell-cycle-state cells in the tissue center and in the tissue edge, defined as regions extending ∼200 μm from the tissue center and tissue edge, respectively. (*F*) Density profiles in polar coordinates at t=40 h, showing cells in G1, S/G2/M, and postmitotic cells. (*E*) and (*F*) show the average of 11 independent tissue expansions with the same experimental initial condition, with shaded regions indicating one standard deviation with respect to the mean.

In order to understand cell proliferation regulation, numerous experimental studies have explored how spatial and mechanical constraints within tissues affect different stages of the cell cycle. The cell cycle consists of four main stages: namely, the G1 phase, where cells grow and prepare for DNA replication; the S phase, during which DNA synthesis occurs; the G2 phase, characterized by further cell growth and preparation for mitosis; and finally, the M phase, where cell division takes place. Cells can also exit the cell cycle and enter G0, where they become quiescent. The experimental visualization of cell cycle stages can be achieved via the widely used FUCCI cell-cycle marker (10), which consists of red and green fluorescent proteins that are fused to proteins Cdt1 and geminin, respectively. Cdt1 exhibits elevated levels during the G0/G1 phase and decreased levels throughout the remaining cell cycle stages, whereas geminin shows high expression during the S, G2, and M phases, allowing thus to distinguish between these different stages; see Fig. 1. Several extensions of the FUCCI system exist now (11); for instance, FUCCI4 allows for the simultaneous visualization of the G1, S, G2, and M phases (12).

Experimental studies of cell migration are often performed in epithelia due to their strong cell-cell adhesion that gives rise to collective and cohesive motion. Moreover, they play a fundamental role in multicellular organisms as they serve as protective layers for various body surfaces and organs. Epithelial cell proliferation is regulated by mechanical forces, which can accelerate, delay, arrest, or reactivate the cell cycle. In particular, extensive research has focused on the G1-S boundary, revealing that intercellular tension can favor this transition (13), whereas tissue pressure can halt progression based on crowding (2).

More generally, the extracellular regulation of switches from G0 and G1, and within substages of G1, has been well known for many years (14). However, and contrary to initial assumptions, cells also have the ability to regulate progression through stages of the cell cycle after the G1-S transition in response to external cues. These external signals might involve not only mechanical forces (15) but also nutrients and growth factors (16,17). In epithelia, this question was explored recently by Donker et al. (18), revealing a mechanical checkpoint in G2 that controls cell division. In particular, this checkpoint allows cells to regulate progression through G2, via sensing of local density, explaining why dense regions in epithelia contain groups of cells that are temporarily halted in G2.

Experimental studies employing FUCCI and variations of it have thus successfully linked mechanical constraints to cell cycle progression. These studies have employed qualitative analysis, direct measurements of cell-cycle-stage durations (18), or metrics associated with cell cycle progression, such as cell area (2), and geminin/Cdt1 or EdU signals (20,21). However, these approaches omit a quantitative comparison between model and data, hence limiting the depth of mechanistic insights that can be derived.

Here, we present a quantitative investigation into the mechanical regulation of cell cycle progression by sensing of local tissue density. First, we construct a mathematical model of cell cycle dynamics that accurately captures the impact of tissue crowding on cell cycle progression. By combining minimal mathematical modeling, Bayesian inference, and recent experimental data (19), we provide further evidence, consistent with previous experimental studies (2,18), that density-dependent effects operate throughout the cell cycle and together serve as a regulating mechanism for the growth of epithelial tissues. Our work thus constitutes a systematic approach toward the quantification of density-dependent effects regulating cell cycle progression. Moreover, the obtained parameter estimates reveal an explicit relation between the duration of different cell cycle stages and tissue density, which is consistent with the experimental measurements of Donker et al. (18).

## Materials and Methods

### Mathematical models of cell cycle dynamics

We build on the model proposed by Vittadello et al. (7) to describe two cell populations, $\rho_{1}\left(x,t\right)$ and $\rho_{2}\left(x,t\right)$, in different stages of the cell cycle. We represent by $\rho_{1}$ the density of cells that are in G0/G1, whereas $\rho_{2}$ gives the density of cells in the S/G2/M phases of the cell cycle; see Fig. 1. In the model, cell motility is described via linear diffusion, with a diffusion constant D>0 for both cell populations (22). In order to effectively capture density-dependent effects controlling cell cycle progression, we assume that the transitions between different cell cycle stages are regulated by two “crowding functions”, f(ρ) and g(ρ), which depend on the total cell density $\rho =\rho_{1}+\rho_{2}$. In particular, the transition rate from G1 to S is given by $k_{1}f\left(\rho \right)$, whereas the division rate (from S/G2/M to G1) is given by $k_{2}g\left(\rho \right)$, where $k_{1},k_{2}>0$ are intrinsic rates of cell cycle progression. With this, the model reads

(1) $$\begin{array}{cc} \partial_{t}\rho_{1} & =D\Delta \rho_{1}-k_{1}\rho_{1}f\left(\rho \right)+2k_{2}\rho_{2}g\left(\rho \right)\text{,}\\ \partial_{t}\rho_{2} & =D\Delta \rho_{2}+k_{1}\rho_{1}f\left(\rho \right)-k_{2}\rho_{2}g\left(\rho \right)\text{,} \end{array}$$

where the factor of 2 in the equation for $\rho_{1}$ represents cell division into two daughter cells, and $\Delta =\sum_{i=1}^{d}\partial_{x_{i}}^{2}$ is the Laplacian operator in dimension *d*. These equations are solved first in polar coordinates (assuming radial symmetry in two spatial dimensions, d=2) to describe epithelial tissue expansion experiments and then in one spatial dimension (d=1) to study traveling wave behavior and the impact of tissue crowding on cell migration phenomena.

In order to accurately capture density-dependent effects regulating cell cycle progression, we assume that *f* and *g* are nonincreasing functions of the total density ρ. Again, this is motivated by the experimental observations of Streichan et al. (2) and Donker et al. (18). Furthermore, we assume f(0)=g(0)=1, so that $k_{1}$ and $k_{2}$ represent density-independent transition rates. Note that setting f=g≡1 gives rise to an exponential growth model (i.e., no dependence on density). On the other hand, choosing f≡1 and $g\left(\rho \right)={\left(1-\rho /K\right)}_{+}$, we recover the Vittadello et al. model (7). Here, we assume that f(ρ) and g(ρ) decrease linearly with the total cell density so that

(2) $$f\left(\rho \right)={\left(1-\frac{\rho }{K_{1}}\right)}_{+},\;g\left(\rho \right)={\left(1-\frac{\rho }{K_{2}}\right)}_{+}\text{,}$$

where $K_{1},K_{2}>0$ are constants controlling the duration of G1 and the S/G2/M phases, respectively, and ${\left(z\right)}_{+}=\max\left(z,0\right)$. The specific form of these “crowding functions” is chosen here for simplicity, although other functions sharing the same properties show similar qualitative behavior.

We follow a Bayesian approach (8,23,24,25) to calibrate the model given in Eq. (1). In particular, given experimental measurements of the cell densities ${\left\{\rho_{k}^{D}\left(x_{i},t_{j}\right)\right\}}_{i,j}$ for k=1,2, and a vector of model parameters $\theta =\left(D,k_{1},k_{2},K_{1},K_{2}\right)$, we estimate the posterior probability distribution $p\left(\theta |\rho^{D}\right)$, which gives the probability density for the model parameters taking specific values. The posterior distribution, thus, can be used to quantify the uncertainty associated with specific parameter values, given the experimental observation. We refer the reader to the supporting material for more details on Bayesian inference.

## Results

### Tissue expansion experiments

We compare our model predictions to the experiments performed by Heinrich et al. (19) studying the expansion and growth dynamics of a single circular epithelial tissue; see Fig. 1 *B*. In these experiments, MDCK cells expressing the FUCCI markers are cultured in a silicone stencil for 18 h, and after stencil removal, the cell population is allowed to freely expand for 46 h. Given that the average cell cycle duration for MDCK cells is around 16 h, this enables each cell to potentially undergo two to three cell divisions during the experiment. Local densities are then quantified by segmenting the fluorescence images in ImageJ and counting the number of nucleus centroids; see Fig. 1 *C*. Note that postmitotic cells do not fluoresce and appear dark, which makes the FUCCI system unreliable for cell counting. To quantify the density of postmitotic cells, Heinrich et al. used a convolutional neural network to identify nuclei from phase contrast images (26); see (19) for more details. Moreover, and in line with previous work (24), the model takes as initial condition the quantified density profile 10 h after stencil removal, so that the impact of the stencil on the dynamics is reduced. Note that after this time, cell densities near the tissue center are relatively high (∼3500 cells/mm^2^, which corresponds to around 50%–70% of the maximum saturation density for MDCK (19,24)), and a fraction of cells in this region are likely to be found in a quiescent state due to contact inhibition of locomotion and proliferation (27).

The experiments by Heinrich et al. (19) reveal a higher density of cells in G0/G1 at the center of the tissue, where the total cell density is also higher; see Fig. 1 *D* and *E*. The tissue edge, in contrast, is characterized by a larger number of cells that are preparing to divide (green) or are directly postmitotic (gray). This agrees with previous observations of epithelial cells, which are known to control progression from G1 to S in response to spatial constraints (2). Note, however, that the density of cells in S/G2/M in the tissue center is low but nonzero, even at later times in the experiment—as observed also by Donker et al. (18); see Fig. 1 *D–F*.

For the sake of simplicity, here, we consider postmitotic cells (gray in Fig. 1) and cells in G0/G1 as one single cell population. Quantifying postmitotic cell density is crucial in order to estimate both $K_{1}$ and $K_{2}$ in Eq. (2), given that these parameters are measures of contact inhibition of proliferation, typically associated with regions of higher cell density (28).

**Figure 2:**
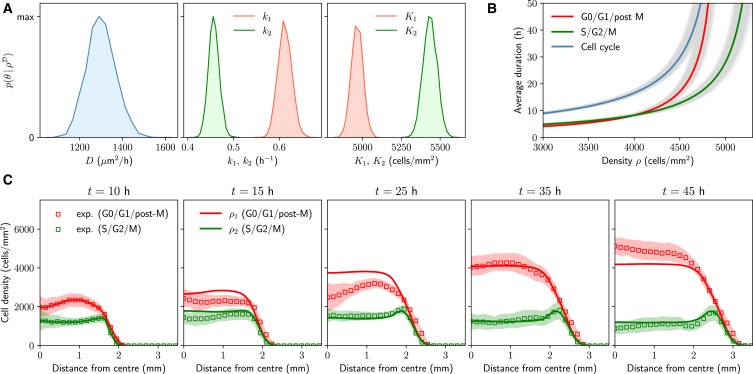
Density-dependent effects regulate cell cycle dynamics in epithelial tissue expansion experiments (19). Parameter estimation and model-data comparison for the model given by Eqs. (1) and (2). (*A*) Univariate marginal posterior distributions for the model parameters. Posterior modes are given by $\left(D,k_{1},k_{2},K_{1},K_{2}\right)=(1300\pm 66$ μm^2^/h, 0.612±0.015 h^−1^, 0.457±0.011 h^−1^, 4965±38 cells/mm^2^, 5435±45 cells/mm^2^), where errors correspond to one standard deviation. (*B*) Estimated duration of the G0/G1/post M (*red*) and S/G2/M (*green*) phases, as well as the whole cell cycle (*black*), as a function of cell densities. Solid lines correspond to posterior modes, and shaded regions are obtained sampling from the posterior distribution. (*C*) Comparing data and model predictions. Squares represent the estimated cell density obtained by averaging 11 experimental realizations, which we use to calibrate the model. Shaded regions denote one standard deviation with respect to the mean; see Fig. S2 for confidence intervals in the model predictions. Numerical simulations in polar coordinates were obtained by using the posterior modes as parameter values and no-flux boundary conditions; for details on the numerical scheme, we refer to the supporting material. In order to minimize the effects of the stencil removal on cell behavior, the initial condition corresponds to the experimental density profile 10 h after stencil removal.

To calibrate the model, we fit to the estimated cell density obtained by averaging eleven experimental realizations. We show the univariate marginal posterior distributions corresponding to the model parameters in Fig. 2 *A*, confirming that all model parameters are practically identifiable. In particular, all marginal posteriors show well-defined and unimodal distributions, with a relatively narrow variance. For more details on model calibration, we refer to the supporting material (Fig. S1).

The posterior distributions in Fig. 2 *A* are not only useful to inform further model predictions but also give insights into the fundamental mechanisms underlying cell proliferation. In particular, given the intrinsic transition rate from G1 to S, $k_{1}$, and the constant $K_{1}$ in Eq. (2), we can estimate the average duration of the combined G1/post-M phase, for a given fixed density ρ, as $1/k_{1}f\left(\rho \right)=1/\left(k_{1}{\left(1-\rho /K_{1}\right)}_{+}\right)$. Analogously, the estimated average duration of the S/G2/M phases is given by $1/k_{2}g\left(\rho \right)=1/\left(k_{2}{\left(1-\rho /K_{2}\right)}_{+}\right)$. Note, however, that these are only estimates of the timescales associated with different cell cycle stages. Put together, these estimates predict for a range of densities between 4000 and 4500 cells/mm^2^, a population doubling time of 14–20 h. In Fig. 2 *B*, we plot these timescales as a function of the density ρ, observing how the duration of the different cell cycle stages increases with density. These results confirm again, in line with previous experimental measurements (2,18), that cell cycle dynamics are tightly regulated by density-dependent effects. In particular, these estimates are consistent with the experimental measurements of Donker et al. (18)—taking into account that the initial cell densities in our data sets are around ρ∼3500 cells/mm^2^. At very low densities, however, our estimates predict a relatively short cell cycle duration. This suggests that the shape of the crowding functions *f* and *g* might be closer to a constant function in this regime.

In Fig. 2 *C*, we show numerical solutions of the model (Eqs. 1 and 2), taking the posterior modes as parameter values. These confirm that the model can describe cell cycle dynamics inside expanding epithelial tissues. Notably, the model captures the tissue expansion speed, as well as the S/G2/M density peak near the edge of the tissue, which results from density-dependent effects regulating the cell cycle. We also note that this type of density profile is possible in the model when crowding-dependent effects are stronger in the early stages of the cell cycle (G1/post M) and weaker in the latter ones (S/G2/M). In terms of Eq. (2), this requires having $K_{1}<K_{2}$, which is correctly identified from the data.

We observe that the model overestimates the experimental density for early times of the experiment and, as a result of the model fit, underestimates it at later times. This is likely due to the transient behavior that cells exhibit immediately after stencil removal (29,30), which could have an impact on cell behavior even after the first 10 h of expansion, as suggested also in previous studies (24). However, we emphasize that tissue edge motion can be well described by the model.

A similar behavior is reported when the model is compared with a second set of experiments performed by Heinrich et al. (19). In this case, we use the obtained parameter estimates to describe the expansion of initially smaller epithelial monolayers (initial diameter ∼1.7 mm). We highlight that the mathematical model can capture the expansion dynamics near the tissue edge as well as the expansion speed (see Fig. S3), even though model parameters were inferred from the large tissue expansions.

### Tissue colonization experiments

Our model, together with the experiments of Heinrich et al. (19), reveals the intrinsic connection between tissue crowding and cell cycle progression, showcasing how this interplay can give rise to spatiotemporal patterns of cell proliferation in growing tissues. Next, we show how the model can be used to study and describe similar patterns observed in several other experimental studies using FUCCI and variants of it.

**Figure 3:**
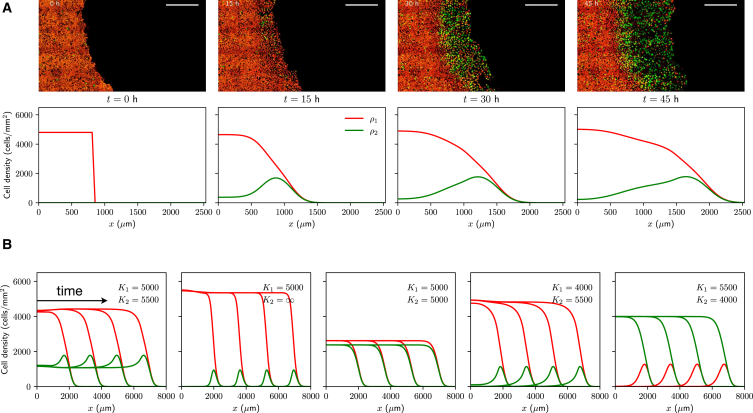
Cell cycle regulation by tissue crowding impacts cell migration. (*A*) Comparison with the tissue colonization experiments of Streichan et al. (2) (top row, adapted with permission). Scale bars correspond to 500 μm. Bottom row shows numerical solutions of Eq. (1) on a one-dimensional domain of length 3000 μm with no-flux boundary conditions, and initial conditions are as follows: $\rho_{1}\left(x,0\right)=4800$ cells/mm^2^ for x<850 μm and $\rho_{1}\left(x,0\right)=0$ cells/mm^2^ otherwise; $\rho_{2}\left(x,0\right)=0$. Parameter values correspond to the posterior modes in Fig. 2. (*B*) Traveling wave solutions of Eq. (1) for different values of $K_{1}$ and $K_{2}$ and at time points t=50,100,150,200 h. Units of $K_{1}$ and $K_{2}$ are cells/mm^2^. Initial conditions: $\rho_{1}\left(x,0\right)=\rho_{2}\left(x,0\right)=500$ cells/mm^2^ for x<850 μm, and $\rho_{1}\left(x,0\right)=\rho_{2}\left(x,0\right)=0$ cells/mm^2^ otherwise. In all cases, all parameters except for $K_{1}$ and $K_{2}$ are fixed (taken from posterior modes).

Streichan et al. (2) show, using a tissue barrier assay, how the cell cycle can be reactivated by allowing cells to migrate and colonize free space; see top row in Fig. 3 *A*. These experiments are initialized by growing MDCK-2 FUCCI cells in the G0/G1 phase within a removable barrier. After barrier removal, the tissue quickly colonizes the available space, and cells behind the barrier, which were initially in G0/G1, reactivate their cycle by entering S phase. On the other hand, cells located further behind the barrier remain at high density and do not progress through the cell cycle.

By solving numerically Eq. (1) on a one-dimensional domain—see bottom row in Fig. 3 *A*—we immediately observe how a model accounting for density-dependent regulation predicts similar behavior to that observed experimentally (note that the experimental images from Streichan et al. (2) do not show postmitotic cells, which appear dark in the FUCCI system, and that the total cell density, including postmitotic cells, was used to estimate the parameters in the model). In particular, and as inferred from the experimental data of Heinrich et al. (19), the calibrated model predicts that crowding-dependent effects have a greater impact at the G1-S transition, compared with the S/G2/M phases. In terms of the model and the choice of crowding functions (Eq. 2), this once again requires $K_{1}<K_{2}$.

### Cell cycle regulation and cell migration

Given that assuming $K_{1}<K_{2}$ seems necessary in order to obtain biologically realistic model predictions, what role do crowding constraints play in shaping cell migration patterns? We explore this question by varying the values of $K_{1}$ and $K_{2}$ in Eq. (2) (Fig. 3 *B*). First, we observe that the density of S/G2/M peaks near the tissue edge when $K_{2}>K_{1}>0$ and remains low in the tissue bulk as long as $K_{2}\gg K_{1}$. However, for $K_{2}∼K_{1}$, the height of this peak decreases, and the fraction of S/G2/M cells in the tissue bulk increases. On the other hand, when we assume a higher influence of density during S/G2/M relative to G1/post-M ($K_{2}<K_{1}$), we observe that the tissue center shows a higher fraction of cells in S/G2/M, in contrast with previously reported observation of contact inhibition of proliferation (27).

The numerical solutions in Fig. 3 *B* suggest that low-density initial conditions lead to traveling wave solutions in one spatial dimension: $\rho_{1}\left(x-ct\right)$, $\rho_{2}\left(x-ct\right)$, with c>0 being the wave speed, and *x* denoting the spatial coordinate. Standard arguments—see supporting material—predict the existence of a minimum traveling wave speed in terms of only three model parameters:

(3) $$c_{\min}=\sqrt{2D\left(-k_{1}-k_{2}+\sqrt{k_{1}^{2}+k_{2}^{2}+6k_{1}k_{2}}\;\right)}\;\text{.}$$

Interestingly, this suggests that the invasion speed is independent of cell cycle regulation and only depends on cell motility (*D*) and the intrinsic, density-independent growth rates ($k_{1}$ and $k_{2}$). However, we highlight that, as shown in the figure, crowding constraints play an important role in shaping collective migration patterns.

The expression for the minimum traveling wave speed facilitates a comparison between the two-stage model proposed here (Eq. 1) and conventional single-population models of cell migration of the form

$$\partial_{t}\rho =D\Delta \rho +r\rho F\left(\rho \right)\text{,}$$

where *F* is a nonincreasing function satisfying F(0)=1. The intrinsic growth rate of the population, *r*, is related to the intrinsic rates of cell cycle progression, $k_{1}$ and $k_{2}$, via $r^{-1}=k_{1}^{-1}+k_{2}^{-1}$. When $4r/\left(k_{1}+k_{2}\right)\ll 1$, Eq. (3) can be approximated by

$$c_{\min}∼2\sqrt{Dr},\;r=\frac{k_{1}k_{2}}{k_{1}+k_{2}}\text{,}$$

which agrees with the prediction of the well-known Fisher- Kolmogorov-Petrovsky-Piskunov equation (F(ρ)=1−ρ/K for a maximum cellular density K>0) in one spatial dimension. Using the estimated parameter values, we obtain $4r/\left(k_{1}+k_{2}\right)∼0.98$, and in this case, Eq. (3) predicts a minimum traveling wave speed of $c_{\min}∼33$ μm/h, whereas the Fisher- Kolmogorov-Petrovsky-Piskunov approximation yields $c_{\min}∼26$ μm/h; both of them within the measured values by Heinrich et al. (19).

A better comparison with the two population model can be obtained by setting rF(ρ)=λ(ρ), where λ(ρ) is the dominant eigenvalue of the growth matrix

$$\left(\begin{array}{cc} -k_{1}f\left(\rho \right) & 2k_{2}g\left(\rho \right)\\ k_{1}f\left(\rho \right) & -k_{2}g\left(\rho \right) \end{array}\right)\text{,}$$

as given by Eq. (1). In this case,

$$c_{\min}=2\sqrt{D\lambda \left(0\right)}\text{,}$$

where $\lambda \left(0\right)=\left(-k_{1}-k_{2}+\sqrt{k_{1}^{2}+k_{2}^{2}+6k_{1}k_{2}}\right)/2$, agreeing with the prediction from Eq. (3).

**Figure 4:**
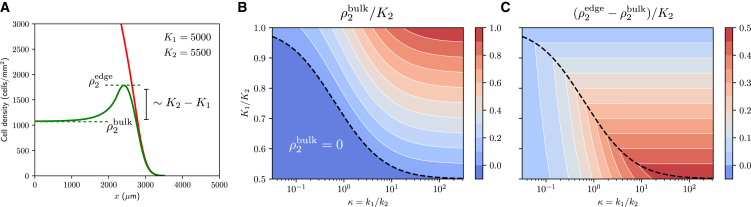
Cell cycle transition rates ($k_{1},k_{2}$) and crowding constraints ($K_{1},K_{2}$) determine cell proliferation patterns in growing tissues. (*A*) Schematic of traveling wave solutions near the tissue edge. (*B* and *C*) Approximated S/G2/M cell densities at the tissue edge and tissue bulk as a function of the ratios $\kappa =k_{1}/k_{2}$ and $K_{1}/K_{2}$. The black dashed line corresponds to the curve $K_{1}/K_{2}=\alpha \left(\kappa \right)=2/\left(\sqrt{\kappa^{2}+6\kappa +1}-\kappa +1\right)$.

More generally, we noted that $c_{\min}$ does not depend on the choice of crowding functions *f* and *g*; however, crowding constraints have an impact on the observed migration patterns (Fig. 3 *B*). To understand how growth and cell cycle regulation lead to the patterns observed experimentally, we investigate traveling wave solutions in a simplified version of our model, utilizing the same parameters as in Eqs. (1) and (2) (see supporting material). In particular, we set $f\left(\rho \right)=H\left(K_{1}-\rho \right)$, and $g\left(\rho \right)=H\left(K_{2}-\rho \right)$, where H(·) denotes the Heaviside function. This reduced model does not accurately approximate the model presented in Eqs. (1) and (2), but nonetheless, it captures the same qualitative behavior, and hence, we expect that the relevant phenomena show similar dependence with respect to model parameters (see Fig. S4). The analysis of traveling wave solutions for this simpler model suggests that the density of S/G2/M cells in the tissue bulk, $\rho_{2}^{\text{bulk}}$, only depends on the ratio of cell cycle progression rates, $\kappa =k_{1}/k_{2}$, and on the ratio of densities associated with crowding constraints, $K_{1}/K_{2}$, (Fig. 4). In particular, we obtain

$$\frac{\rho_{2}^{\text{bulk}}}{K_{2}}∼\left\{ \begin{array}{cc} \frac{K_{1}}{K_{2}}\frac{1}{\alpha \left(\kappa \right)}-1, & K_{1}/K_{2}>\alpha \left(\kappa \right)\;;\\ 0, & K_{1}/K_{2}\leq \alpha \left(\kappa \right)\;; \end{array}\right.$$

where $\alpha \left(\kappa \right)=2/\left(\sqrt{\kappa^{2}+6\kappa +1}-\kappa +1\right)$. A similar dependence with respect to the model parameters is observed numerically for the model given by Eqs. (1) and (2) (Fig. S4). For our estimated parameters, the expression above predicts $\rho_{2}^{\text{bulk}}/K_{2}∼0.3$, which is consistent with experimental observations. We also highlight that, as long as $K_{1}<K_{2}$, and $k_{1}$ and $k_{2}$ are of a similar order of magnitude, this expression predicts that the number of cells in S/G2/M in the tissue bulk will be small in comparison to the number of cells in G1/post-M (Fig. 4 *B*). In particular, note that $\rho_{2}^{\text{bulk}}\to 0$ as $K_{2}\to \infty$.

Interestingly, the traveling wave analysis also reveals that, when $\rho_{2}^{\text{bulk}}>0$, the difference in S/G2/M cell density between the tissue bulk, $\rho_{2}^{\text{bulk}}$, and the tissue edge, $\rho_{2}^{\text{edge}}$, depends only on the difference of densities associated to crowding constraints at the G1-S and G2-M boundaries (Fig. 4 *C*),

$$\rho_{2}^{\text{edge}}-\rho_{2}^{\text{bulk}}∼\left\{ \begin{array}{cc} K_{2}-K_{1}, & K_{1}/K_{2}>\alpha \left(\kappa \right)\;;\\ \rho_{2}^{\text{edge}}, & K_{1}/K_{2}\leq \alpha \left(\kappa \right)\;; \end{array}\right.$$

where

$$\frac{\rho_{2}^{\text{edge}}}{K_{2}}∼\frac{K_{1}}{K_{2}}\frac{1-\alpha \left(\kappa \right)}{\alpha \left(\kappa \right)}\;\text{.}$$

For our estimated parameters, we obtain $\rho_{2}^{\text{edge}}-\rho_{2}^{\text{bulk}}∼500$ cells/mm^2^, again consistent with the experimental observations. These analytical expressions confirm the impact of density-dependent effects on cell migration and suggest that differences in the regulation of cell cycle stages contribute to the emergence of cell proliferation patterns.

### Density-dependent effects and experimental design

**Figure 5:**
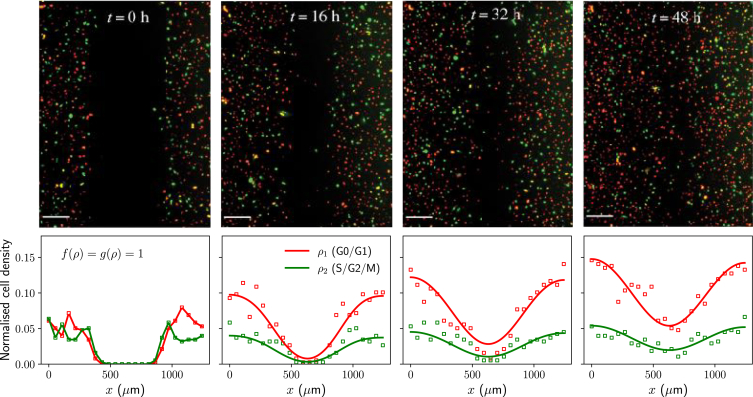
Absence of density-dependent effects in a low-density scratch assay experiment (1205Lu melanoma cells). In this case, an exponential growth model can reproduce the experimental data. Density is normalized by using the theoretical maximum density corresponding to hexagonal close packing of cells (7). Top row is adapted from (8), with scale bars corresponding to 200 μm. Numerical solutions of Eq. (1) with f(ρ)=g(ρ)=1 on a one-dimensional domain. Parameters are estimated using the experimental data from (8); see Fig. S6 for posterior distributions.

We have demonstrated that our model can be calibrated to experimental data collected by Heinrich et al. (19) to provide confident estimates of all parameters and, from there, used to extract and quantify crowding constraints regulating the cell cycle. An obvious question to ask is whether the model parameters could also be confidently estimated from other data sets, in particular where the cell density remains much lower and the impact of tissue crowding is reduced. To explore this question, we attempt to estimate the model parameters (including $K_{1}$ and $K_{2}$) using data from a low-density scratch assay with 1205Lu melanoma cells; see Fig. 5. We highlight that melanoma cells are highly metastatic and often display uncontrolled and invasive migration, in contrast to the highly collective and regulated movement exhibited by epithelial cells. In this experiment, tissues are seeded at an initial density of ∼400 cells/mm^2^ (5% of the theoretical maximum packing density (7)), and data are collected every 16 h, over 2 full days, allowing cells to potentially undergo one to two cell cycles. The posterior distributions obtained for the different model parameters reveal estimates for *D*, $k_{1}$, and $k_{2}$ that are consistent with previous studies (8). However, the low experimental densities do not allow for the quantification of density-dependent effects; the parameters $K_{1}$ and $K_{2}$ cannot be estimated with any degree of confidence (see Fig. S5). This nonidentifiability of $K_{1}$ and $K_{2}$ suggests the use of a simpler model, which assumes that cell cycle progression is independent of density-dependent effects (f(ρ)=g(ρ)=1) and hence is only valid in the low-density regime. Indeed, when calibrated to data from the low-density scratch assay, it provides accurate parameters estimates (Fig. S6) and an excellent agreement with the experimental data; see Fig. 5. This result clearly illustrates both the key role that mathematical modeling can play in the experimental design process and the importance of considering parameter identifiability in the process of model construction.

## Discussion

In this work, we have presented a new mathematical model of cell migration with cell cycle dynamics that captures and quantifies cell cycle regulation by sensing levels of tissue crowding. In line with previous experimental studies, by combining minimal modeling and Bayesian inference, we confirm that cell cycle progression is monitored via crowding constraints (2,18), and we present a systematic approach toward the quantification of interactions regulating cell proliferation. Our model is capable of quantifying cell cycle data from experiments using the FUCCI system, and it enables the extraction of mechanistic insights into how individual cells regulate proliferation based on population-level measures.

The model presented here offers several applications to further our understanding of cell-cell interactions in cell proliferation. In particular, our model presents a systematic way to quantify the impact of drugs and gene knockouts/knockdowns interfering with cell proliferation. By using parameter estimation techniques, applied to different experimental data sets, we can gain insights into the regulatory roles of specific genes in the cell cycle. Another possible application concerns the study of cell migration in biomaterials incorporating cadherin proteins, which have recently been shown to slow down cell cycle dynamics (20). Furthermore, generalizations of the FUCCI system could allow for a finer representation of the different cell cycle stages; for instance, FUCCI4 (12) allows for the simultaneous visualization of the four stages of the cell cycle. In line with these methodologies, extensions of our model (Eq. 1) to multistage cell populations are straightforward and could enable a more exhaustive explanation of the role of spatial constraints across all four cell cycle stages (18).

In the case of Heinrich et al.’s experiments (19), the excellent imaging quality allowed us to perform an accurate quantification of the cellular density profiles. This, in turn, facilitated model development and the subsequent inference of model parameters from the data, with the estimated parameters showing a low uncertainty. Although the parameter identifiability of such mathematical models can be evaluated a priori under the assumption of infinite ideal data (31,32), biologically realistic data sets are finite and often contain a significant level of noise, which can, in certain instances, constrain the ability to confidently estimate model parameters. More generally, and as we have illustrated, practical constraints in the experimental data often relate to the level of model complexity that can be inferred from experiments and the confidence in model parameter estimates.

Continuum models are a widely adopted approach for describing cell migration. However, these models come with limitations: they tend to neglect local structure, especially in situations involving multiple cell populations. Such local structure can be observed in Fig. 1 *C* and *D*, which shows some degree of local correlation in the cell phases; however, this phenomenon is lost when averaging radially to obtain the density profiles in Fig. 1 *E*. Agent-based models (21,33,34) can help mitigate some of these issues, by providing more understanding of the generation and maintenance of spatial structure, but at the cost of increased computational times for simulation and inference, additional model parameters, and limited analytical tractability. We emphasize, however, that cell cycle dynamics appear to be globally desynchronized, as observed in previous studies (35), and so our differential equation-based model remains appropriate for this study, where the data are generated by averaging over a number of experimental replicates.

The model presented here is minimal in the sense that it assumes that cell movement is random, and it ignores basic cell-cell interactions that are typical of epithelial cell migration such as cell-cell adhesion. Although local cell density is likely to have an impact on cell motility (19), previous work shows that for individual expanding epithelial tissues, the linear diffusion model provides a good approximation (24). Note, however, that it is important to account for population pressure and its impact on cell movement when considering tissue-tissue interactions (36). Additional research is needed to determine whether more complicated models (37,38) incorporating cell-cell adhesion and other basic interactions offer deeper mechanistic understanding. Moreover, the model given in Eq. (1) assumes that at low densities, the duration of each of the cell cycle stages follows an exponential distribution. Although this assumption contradicts experimental observations (39, 40) and can be mitigated by representing the cell cycle as a multistage process (9,41), such models break the cell cycle into a very large number of stages, limiting the potential for calibration to experimental data. Additional investigation is required to understand the extent to which more complicated models can provide further insights into how cells coordinate proliferation and migration to give rise to complex collective behaviors. For example, in the context of the cell cycle, an option is to explicitly incorporate cell cycle stage via the use of an age-structured model (42) that includes density-dependent regulation. Our results indicate that adopting a quantitative approach (43), which carefully examines quantitative data through the lens of mathematical modeling and Bayesian inference, can help provide answers to this question.

## Data and code availability

Code to solve the model and to perform the parameter estimation is available on Github: https://github.com/carlesfalco/InferenceCellCyclePDE. Data used to calibrate the model can also be found on Github and in (19). Scratch assay data is taken from (8).

## Author contributions

C.F. and R.E.B. conceived the original idea. C.F. created the code, carried out the analysis, and wrote the manuscript with input from R.E.B. J.A.C. helped supervise the project. D.J.C. provided the experimental data and aided in interpreting the results. All authors gave final approval for publication.

## Supporting Material

Document S1. Figures S1–S6

Document S2. Article plus Supporting Material

## References

[1] Jorgensen P., Tyers M. (2004). How cells coordinate growth and division. Curr. Biol..

[2] Streichan S.J., Hoerner C.R., Hufnagel L. (2014). Spatial constraints control cell proliferation in tissues. Proc. Natl. Acad. Sci. USA.

[3] Massagué J. (2004). G1 cell-cycle control and cancer. Nature.

[4] McClatchey A.I., Yap A.S. (2012). Contact inhibition (of proliferation) redux. Curr. Opin. Cell Biol..

[5] Otto T., Sicinski P. (2017). Cell cycle proteins as promising targets in cancer therapy. Nat. Rev. Cancer.

[6] Gupta V.K., Chaudhuri O. (2022). Mechanical regulation of cell-cycle progression and division. Trends Cell Biol..

[7] Vittadello S.T., McCue S.W., Simpson M.J. (2018). Mathematical models for cell migration with real-time cell cycle dynamics. Biophys. J..

[8] Simpson M.J., Baker R.E., Maclaren O.J. (2020). Practical parameter identifiability for spatio-temporal models of cell invasion. J. R. Soc. Interface.

[9] Gavagnin E., Ford M.J., Yates C.A. (2019). The invasion speed of cell migration models with realistic cell cycle time distributions. J. Theor. Biol..

[10] Sakaue-Sawano A., Kurokawa H., Miyawaki A. (2008). Visualizing spatiotemporal dynamics of multicellular cell-cycle progression. Cell.

[11] Ridenour D.A., McKinney M.C., Kulesa P.M. (2012). CycleTrak: a novel system for the semi-automated analysis of cell cycle dynamics. Dev. Biol..

[12] Bajar B.T., Lam A.J., Lin M.Z. (2016). Fluorescent indicators for simultaneous reporting of all four cell cycle phases. Nat. Methods.

[13] Uroz M., Wistorf S., Trepat X. (2018). Regulation of cell cycle progression by cell-cell and cell-matrix forces. Nat. Cell Biol..

[14] Pardee A.B. (1989). G1 events and regulation of cell proliferation. Science.

[15] Godard B.G., Heisenberg C.-P. (2019). Cell division and tissue mechanics. Curr. Opin. Cell Biol..

[16] McKeown C.R., Cline H.T. (2019). Nutrient restriction causes reversible G2 arrest in Xenopus neural progenitors. Development.

[17] Celora G.L., Bader S.B., Byrne H.M. (2022). A DNA-structured mathematical model of cell-cycle progression in cyclic hypoxia. J. Theor. Biol..

[18] Donker L., Houtekamer R., Gloerich M. (2022). A mechanical G2 checkpoint controls epithelial cell division through E-cadherin-mediated regulation of Wee1-Cdk1. Cell Rep..

[19] Heinrich M.A., Alert R., Cohen D.J. (2020). Size-dependent patterns of cell proliferation and migration in freely-expanding epithelia. Elife.

[20] Suh K., Cho Y.K., Cohen D.J. (2024). E-cadherin biointerfaces reprogram collective cell migration and cell cycling by forcing homeostatic conditions. Cell Rep.

[21] Höllring K., Nuić L., Smith A.-S. (2023). Capturing the mechanosensitivity of cell proliferation in models of epithelium. bioRxiv.

[22] Vittadello S.T., McCue S.W., Simpson M.J. (2020). Examining go-or-grow using fluorescent cell-cycle indicators and cell-cycle-inhibiting drugs. Biophys. J..

[23] Hines K.E., Middendorf T.R., Aldrich R.W. (2014). Determination of parameter identifiability in nonlinear biophysical models: A Bayesian approach. J. Gen. Physiol..

[24] Falcó C., Cohen D.J., Baker R.E. (2023). Quantifying tissue growth, shape and collision via continuum models and Bayesian inference. J. R. Soc. Interface.

[25] Schälte Y., Fröhlich F., Hasenauer J. (2023). pyPESTO: a modular and scalable tool for parameter estimation for dynamic models. Bioinformatics.

[26] LaChance J., Cohen D.J. (2020). Practical fluorescence reconstruction microscopy for large samples and low-magnification imaging. PLoS Comput. Biol..

[27] Puliafito A., Hufnagel L., Shraiman B.I. (2012). Collective and single cell behavior in epithelial contact inhibition. Proc. Natl. Acad. Sci. USA.

[28] Warne D.J., Baker R.E., Simpson M.J. (2017). Optimal quantification of contact inhibition in cell populations. Biophys. J..

[29] Jin W., Shah E.T., Simpson M.J. (2016). Reproducibility of scratch assays is affected by the initial degree of confluence: Experiments, modelling and model selection. J. Theor. Biol..

[30] Jin W., Shah E.T., Simpson M.J. (2017). Logistic proliferation of cells in scratch assays is delayed. Bull. Math. Biol..

[31] Renardy M., Kirschner D., Eisenberg M. (2022). Structural identifiability analysis of age-structured PDE epidemic models. J. Math. Biol..

[32] Browning A.P., Taşcă M., Baker R.E. (2024). Structural identifiability analysis of linear reaction–advection–diffusion processes in mathematical biology. Proc. Royal Soc. A.

[33] Klowss J.J., Browning A.P., Simpson M.J. (2022). A stochastic mathematical model of 4D tumour spheroids with real-time fluorescent cell cycle labelling. J. R. Soc. Interface.

[34] Carpenter L.C., Pérez-Verdugo F., Banerjee S. (2024). Mechanical control of cell proliferation patterns in growing epithelial monolayers. Biophys. J..

[35] Nowak C.M., Quarton T., Bleris L. (2023). Impact of variability in cell cycle periodicity on cell population dynamics. PLoS Comput. Biol..

[36] Heinrich M.A., Alert R., Cohen D.J. (2022). Self-assembly of tessellated tissue sheets by expansion and collision. Nat. Commun..

[37] Carrillo J.A., Murakawa H., Trush O. (2019). A population dynamics model of cell-cell adhesion incorporating population pressure and density saturation. J. Theor. Biol..

[38] Falcó C., Baker R.E., Carrillo J.A. (2022). A local continuum model of cell-cell adhesion. arXiv.

[39] Smith J.A., Martin L. (1973). Do cells cycle?. Proc. Natl. Acad. Sci. USA.

[40] Weber T.S., Jaehnert I., Carneiro J. (2014). Quantifying the length and variance of the eukaryotic cell cycle phases by a stochastic model and dual nucleoside pulse labelling. PLoS Comput. Biol..

[41] Yates C.A., Ford M.J., Mort R.L. (2017). A multi-stage representation of cell proliferation as a Markov process. Bull. Math. Biol..

[42] Kynaston J.C., Guiver C., Yates C.A. (2022). Equivalence framework for an age-structured multistage representation of the cell cycle. Phys. Rev. E.

[43] Liu Y., Suh K., Baker R.E. (2024). Parameter identifiability and model selection for partial differential equation models of cell invasion. J. R. Soc. Interface.

