# Supplementary material for: Quantifying cell cycle regulation by tissue crowding
Source: Biophys J. 2024 May 7;124(6):923–32. doi: 10.1016/j.bpj.2024.05.003 (PMC11947467; doi:10.1016/j.bpj.2024.05.003)
Supplement: Document S1. Figures S1–S6 [file mmc1.pdf]

Biophysical Journal, Volume *124*

Supplemental information

Quantifying cell cycle regulation by tissue crowding

Carles Falcó, Daniel J. Cohen, José A. Carrillo, and Ruth E. Baker

S1 Bayesian parameter estimation

All experimental datasets [2, 8] consist of direct measurements of the density of cells in the G1/post-M, and S/G2/M phases of the cell cycle. We denote these measurements by $\{\rho_1^{\mathcal{D}}(\mathbf{x}_i, t_j), \rho_2^{\mathcal{D}}(\mathbf{x}_i, t_j)\}_{i,j}$. The next step in order to estimate the different model parameters is to assume a so-called error model, which relates experimental measurements with the model predictions given by the solutions of the model: $\rho_1(\mathbf{x}, t), \rho_2(\mathbf{x}, t)$. For simplicity, here we assume that the residuals are independent and normally distributed

$$\rho_1^{\mathcal{D}}(\mathbf{x}_i, t_j) - \rho_1(\mathbf{x}_i, t_j) \overset{\text{iid}}{\sim} N(0, \sigma_1^2)\,,$$
$$\rho_2^{\mathcal{D}}(\mathbf{x}_i, t_j) - \rho_2(\mathbf{x}_i, t_j) \overset{\text{iid}}{\sim} N(0, \sigma_2^2)\,,$$

where σ_1 and σ_2 are parameters to be estimated from the data.

The white noise assumption has the advantage that simple likelihood-based methods can be used for inference. In particular, the log-likelihood of observing the data, given specific model parameters θ, can be written as

$$\ell_{\mathcal{D}}(\theta) = -\frac{1}{2}\sum_{k=1}^{2}\sum_{i,j}\left(\log\left(2\pi\sigma_k^2\right) + \left(\frac{\rho_k^{\mathcal{D}}(\mathbf{x}_i, t_j) - \rho_k(\mathbf{x}_i, t_j)}{\sigma_k}\right)^2\right).$$

While we assume a simplistic noise model to perform the parameter inference, model misspecification and the temporal resolution of the measured data are likely to introduce correlations between residuals [3]. In particular, some degree of correlation might be expected given the model parameters are very well-determined with a relatively small variance – see Fig. 2 in the main text. More recently, a binomial measurement error model has been suggested in order to mitigate some of the inconsistencies of the white noise assumption [9]. Other commonly used error models assume different forms of multiplicative noise, which preserve the positivity of the data [5, 6]. A more comprehensive study of the error model is left as a subject for future investigation.

Maximising the log-likelihood function would give a set of parameters θ^* that we could use to generate further model predictions. However, this approach does not give any information on the associated uncertainty, which here is of particular interest given that the parameters are estimated from noisy experimental data. To explore parameter identifiability, we follow a Bayesian approach, in which uncertainty associated with model parameters (θ) is quantified in a posterior distribution $P(\theta\,|\,\rho^{\mathcal{D}}) = P(\theta\,|\,\rho_1^{\mathcal{D}}, \rho_2^{\mathcal{D}})$. This posterior distribution can be

calculated from Bayes' theorem

$$P(\theta \,|\, \rho^{\mathcal{D}}) \propto P(\rho^{\mathcal{D}} \,|\, \theta)\pi(\theta),$$

where $P(\rho^{\mathcal{D}} \,|\, \theta) = \exp \ell_{\mathcal{D}}(\theta)$ is the likelihood of observing the measured data, and $\pi(\theta)$ is the prior distribution of the parameter vector θ. For the tissue expansion experiments [2], we assume a log-uniform prior on D, k_1, k_2, with bounds: 10^1 μm^2/h $< D < 10^4$ μm^2/h, 10^{-4} h^{-1} $< k_1, k_2 < 10^1$ h^{-1}. This assumption allows us to consider a broad range of orders of magnitude for these parameters, although simpler uniform priors could also be used. For the parameters $K_1, K_2, \sigma_1, \sigma_2$, uniform priors with the following bounds were used: $0 < K_1, K_2 < 20000$ cells/mm^2, $0 < \sigma_1, \sigma_2 < 2000$ cells/mm^2. For the scratch assay data, we follow [8] and assume $\sigma_1 = \sigma_2 = \sigma$, and a uniform prior in all model parameters with the following conservative bounds: $0 < D < 2000$ μm^2/h, $0 < k_1, k_2 < 0.2$ h^{-1}, $0 < K_1, K_2 < 30000$ cells/mm^2, $0 < \sigma < 4000$ cells/mm^2.

We use a Metropolis-Hastings MCMC (Markov chain Monte Carlo) sampler with adaptive proposal covariance to infer the posterior distributions. This is implemented in the parameter estimation toolbox pyPESTO [7]. In the MCMC algorithm, a Markov Chain starts at position θ and accepts a potential move to θ^* with probability $q = \min\{1, P(\theta \,|\, \rho^{\mathcal{D}})/P(\theta^* \,|\, \rho^{\mathcal{D}})\}$. In this way, the Markov chain tends to move towards high values of the posterior distribution, while still allowing for transitions to regions of lower probability in order to move away from local maxima. Figures S1 and S4 show typical MCMC iterations for both sets of experimental data and the corresponding data. We show the obtained stationary posterior distributions in the main text, and in Fig. S4.

S2 Outline of the numerical scheme

We briefly explain the numerical scheme used to solve our model in polar coordinates. For the tissue expansion experiments we assume solutions with radial symmetry: $\rho_1(\mathbf{x}, t) = \rho_1(r, t)$, $\rho_2(\mathbf{x}, t) = \rho_2(r, t)$, where r denotes the distance from the tissue centre. Hence, we can write

$$\Delta \rho_k = \partial_r^2 \rho_k + r^{-1} \partial_r \rho_k, \quad k = 1, 2\,.$$

We use a finite-volume scheme [2] and discretise the domain into a small circle C_0 of radius $r_{1/2} = \delta r/2$, and concentric annuli C_i with inner radii $r_{i-1/2} = (i - 1/2)/\delta r$, for

$i = 1, 2, \ldots, N$. If $\mathbf{x} \in C_i$, we approximate

$$\rho_k(\mathbf{x}, t) \approx \rho_k^i(t) := \frac{1}{|C_i|} \int_{C_i} \rho_k \,, \quad i = 0, 1, \ldots, N \,;$$

where $|C_i|$ denotes the volume of C_i. In particular, by integrating the equation for ρ_1 over C_0 we obtain

$$\frac{\mathrm{d}\rho_1^0}{\mathrm{d}t} = \frac{2\pi D}{|C_0|} \int_0^{r_{1/2}} r \left(\partial_r^2 \rho_1 + r^{-1} \partial_r \rho_1\right) \mathrm{d}r - \frac{k_1}{|C_0|} \int_{C_0} \rho_1 f(\rho) + \frac{2k_2}{|C_0|} \int_{C_0} \rho_2 g(\rho) \,,$$

where $|C_0| = \pi r_{1/2}^2$ denotes the area of C_0. The first integral can be calculated exactly to obtain

$$\int_0^{r_{1/2}} r \left(\partial_r^2 \rho_1 + r^{-1} \partial_r \rho_1\right) \mathrm{d}r = r(\partial_r \rho)\big|_{r=r_{1/2}} \,.$$

The last two integrals can be approximated to obtain

$$\frac{\mathrm{d}\rho_1^0}{\mathrm{d}t} = \frac{2\pi D}{|C_0|} r(\partial_r \rho_1)\big|_{r=r_{1/2}} - k_1 \rho_1^0 f(\rho^0) + 2k_2 \rho_2^0 g(\rho^0),$$

where $\rho^0 = \rho_1^0 + \rho_2^0$.

Similarly, we integrate the equation for ρ_1 over C_i, $i \geq 1$, to obtain

$$\frac{\mathrm{d}\rho_1^i}{\mathrm{d}t} = \frac{2\pi D}{|C_i|} \left(r(\partial_r \rho_1)\big|_{r=r_{i+1/2}} - r(\partial_r \rho_1)\big|_{r=r_{i-1/2}} \right) - k_1 \rho_1^i f(\rho^i) + 2k_2 \rho_2^i g(\rho^i),$$

where $|C_i| = \pi(r_{i+1/2}^2 - r_{i-1/2}^2)$ and $\rho^i = \rho_1^i + \rho_2^i$. An analogous set of equations can be obtained for ρ_2 following the same arguments. Finally, we approximate the derivatives $\partial_r \rho$ as

$$(\partial_r \rho_k)_{r=r_{i+1/2}} \approx \frac{\rho_k^{i+1} - \rho_k^i}{\delta r}, \quad k = 1, 2;\ i = 0, 1, \ldots, N.$$

We solve the resulting differential equations using a fourth-order Runge-Kutta method implemented in the `scipy.integrate.ode` class in Python.

S3 Minimum travelling wave speed

We look for travelling solutions in the model given by Eqs. (2) in the main text, in one spatial dimension. We assume that the *crowding functions* $f(\rho)$ and $g(\rho)$ are non-increasing with ρ, and non-negative. In the comoving reference frame, we can write: $\rho_1(x,t) = U_1(z)$, $\rho_2(x,t) = U_2(z)$, where $z = x - ct$, and $c \geq 0$ denotes the wave speed. By denoting $U = U_1 + U_2$, $V_1 = U_1'$, $V_2 = U_2'$, the model reduces to

$$\begin{cases} U_1' &= V_1\,, \\ DV_1' &= -cV_1 + k_1 U_1 f(U) - 2k_2 U_2 g(U)\,, \\ U_2' &= V_2\,, \\ DV_2' &= -cV_2 - k_1 U_1 f(U) + k_2 U_2 g(U)\,, \end{cases} \tag{1}$$

where the primes indicate differentiation with respect to z.

The set of steady states of system (1) consists of the origin $(U_1, V_1, U_2, V_2) = (0,0,0,0)$ and any state of the form $(\gamma, 0, U^* - \gamma, 0)$, with $f(U^*) = g(U^*) = 0$ and $0 \leq \gamma \leq U^*$. Note that whenever $f(\rho)$, $g(\rho) > 0$ for all $\rho \geq 0$, the latter does not exist. As usual with linear diffusion models, the stability of the origin gives a lower bound on the wave speed c. In particular the Jacobian of system (1) at the origin reads

$$\begin{pmatrix} 0 & 1 & 0 & 0 \\ k_1/D & -c/D & -2k_2/D & 0 \\ 0 & 0 & 0 & 1 \\ -k_1/D & 0 & k_2/D & -c/D \end{pmatrix}.$$

The eigenvalues λ_i of the linearized system about this point satisfy the polynomial equation

$$\lambda^4 + \frac{2c}{D}\lambda^3 + \left(\left(\frac{c}{D}\right)^2 - \frac{k_1 + k_2}{D}\right)\lambda^2 - c\frac{k_1 + k_2}{D^2}\lambda - k_1 k_2 = 0\,.$$

By defining

$$\gamma^{\pm} = \left(\frac{c}{D}\right)^2 + \frac{2}{D}\left[k_1 + k_2 \pm \sqrt{k_1^2 + k_2^2 + 6k_1 k_2}\right],$$

the roots of this quartic polynomial can be expressed as

$$\lambda_1^{\pm} = \frac{1}{2}\left(-\frac{c}{D} \pm \sqrt{\gamma^+}\right), \quad \lambda_2^{\pm} = \frac{1}{2}\left(-\frac{c}{D} \pm \sqrt{\gamma^-}\right).$$

We seek biologically realistic solutions with $U_1, U_2 \geq 0$, and hence the eigenvalues must be real. In particular, this demands $\gamma^{\pm} \geq 0$, which establishes the minimum travelling wave speed found in [10]

$$c_{\min} = \sqrt{2D\left(-k_1 - k_2 + \sqrt{k_1^2 + k_2^2 + 6k_1k_2}\right)}\,. \tag{2}$$

By writing

$$k_1^2 + k_2^2 + 6k_1k_2 = (k_1 + k_2)^2 + 4k_1k_2 = (k_1 + k_2)^2 \left[1 + \frac{4k_1k_2}{(k_1 + k_2)^2}\right] ,$$

we observe that when $4k_1k_2/(k_1 + k_2)^2 \ll 1$, the minimum travelling wave speed can be approximated by

$$c_{\min} \approx 2\sqrt{D\frac{k_1k_2}{k_1 + k_2}},$$

which agrees with the minimum speed predicted by the Fisher–Kolmogorov–Petrovsky–Piskunov (FKPP) equation [4].

S4 Study of travelling wave solutions

A commonly used approach to obtain approximate solutions for travelling waves is the so-called Canosa's method [1]. This procedure is a standard singular perturbation technique, and consists of a transformation $y = -z/c$, where $D/c^2 := \varepsilon$ is treated as a small parameter. The first-order perturbation in ε approximates, within a small error, travelling solutions of the well-known FKPP equation, even though in this case ε is not necessarily small [4]. In our case, by using the esimated parameters and a wave speed of 30 μm/h, we obtain $\varepsilon \sim O(1)$. We highlight, however, that the lowest order approximation in ε provides an excellent approximation of the travelling wave – see Fig. 1.

By using the transformation $y = -z/c$, we can write system (1) as

$$\frac{\mathrm{d}U_1}{\mathrm{d}y} - \varepsilon\frac{\mathrm{d}^2U_1}{\mathrm{d}y^2} + k_1U_1f(U) - 2k_2U_2g(U) = 0\,, \tag{3}$$

$$\frac{\mathrm{d}U_2}{\mathrm{d}y} - \varepsilon\frac{\mathrm{d}^2U_2}{\mathrm{d}y^2} - k_1U_1f(U) + k_2U_2g(U) = 0\,. \tag{4}$$

Observe that, given the sign of the transformation $y = -z/c$, we need to impose the following

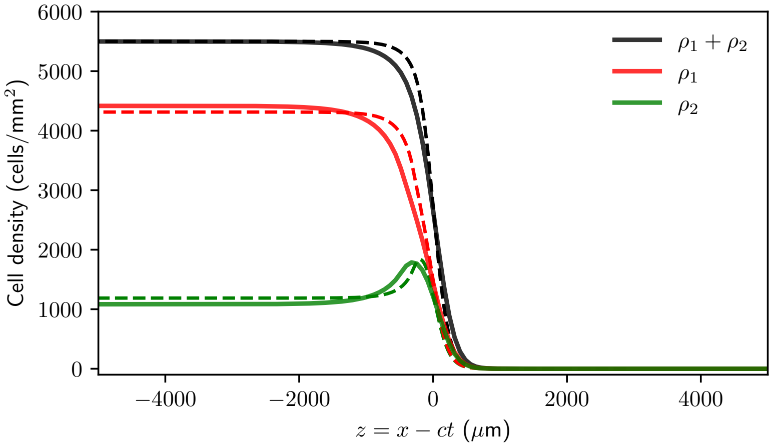

Figure 1: Comparison of travelling wave solutions obtained from the partial differential model (solid lines), given by Eqs. (1) in the main text, and the order $O(1)$ approximation (dashed lines), obtained from solving the ordinary differential equations (5) and (6). Model parameters are taken from posterior distribution modes.

boundary conditions

$$U_1(-\infty) = U_2(-\infty) = 0, \quad U_1(+\infty) = \alpha, \quad U_2(+\infty) = U^* - \alpha\,,$$

with $f(U^*) = g(U^*) = 0$ and $0 \leq \alpha \leq U^*$. For the choice of f and g in the main text ($f(U) = (1 - U/K_1)_+$ and $g(U) = (1 - U/K_2)_+$ with $K_1 < K_2$), we expect $U^* = K_2$ and $\alpha \in [0, K_2]$.

Although the analysis as $\varepsilon \to 0$ looks like a singular perturbation problem, setting $\varepsilon = 0$ gives a valid first-order approximation. This is due to the fact that the nonlinear terms in Eqs. (3) and (4) vanish at both boundaries [4]. Hence, we can look for a regular perturbation expansion in both U_1 and U_2. By denoting the order $O(1)$ solutions as u_1 and u_2 we obtain

$$\frac{\mathrm{d}u_1}{\mathrm{d}y} = -k_1 u_1 f(u) + 2k_2 u_2 g(u)\,, \tag{5}$$

$$\frac{\mathrm{d}u_2}{\mathrm{d}y} = k_1 u_1 f(u) - k_2 u_2 g(u)\,, \tag{6}$$

where $u = u_1 + u_2$. In the figure above (Fig. 1), we compare the approximate solutions obtained by solving this system with the full travelling wave solutions. We highlight that the lowest order approximation provides an excellent approximation of the travelling wave shape.

S4.1 A simplified model

In order to make analytical progress we set f and g to be Heaviside functions: $f(u) = H(K_1 - u)$ and $g(u) = H(K_2 - u)$. This model is not an approximation of the model presented in the main text, but a simplification which preserves the same qualitative behaviour. Hence, we expect that the observed phenomena show similar dependence on the model parameters; this will be numerically confirmed later. In particular, note that this simplified model also describes two density checkpoints, at the G1-S boundary, and during the G2/M phases. The parameters K_1 and K_2, respectively, quantify the cell density associated with these checkpoints.

We rewrite Eqs. (5) and. (6) in terms of the variables (u, u_2),

$$\frac{\mathrm{d}u}{\mathrm{d}y} = k_2 u_2 g(u) \, ,$$
$$\frac{\mathrm{d}u_2}{\mathrm{d}y} = k_1(u - u_2) f(u) - k_2 u_2 g(u) \, .$$

Depending on the relative values of the total cell density, u, and the density checkpoints parameters K_1, K_2, we distinguish three possible cases. As inferred from the experimental data, we assume $K_1 < K_2$.

<u>Tissue edge ($u < K_1 < K_2$).</u> In this region $f(u) = g(u) = 1$ and we can write

$$\frac{\mathrm{d}u}{\mathrm{d}y} = k_2 u_2 \, ,$$
$$\frac{\mathrm{d}u_2}{\mathrm{d}y} = k_1 u - (k_1 + k_2) u_2 \, .$$

The solution at the tissue edge reads

$$u(y) = e^{-(k_1+k_2)y/2} \left[A e^{\gamma y/2} + B e^{-\gamma y/2} \right] \, ,$$
$$u_2(y) = e^{-(k_1+k_2)y/2} \left[\frac{A\left(\gamma - (k_1+k_2)\right)}{2k_2} e^{\gamma y/2} - \frac{B\left(\gamma + (k_1+k_2)\right)}{2k_2} e^{-\gamma y/2} \right] \, ,$$

where A, B are constants to be determined, and $\gamma = \sqrt{(k_1+k_2)^2 + 4k_1k_2}$. Imposing boundary conditions at $y \longrightarrow -\infty$, and noting that $\gamma - (k_1 + k_2) > 0$, we obtain $B = 0$. Hence, for $u < K_1$ both solutions are increasing exponentials. Without loss of generality we set

$U(0) = K_1$, giving $A = K_1$, and thus

$$u(y) = K_1 e^{(\gamma - k_1 - k_2)y/2}\,,$$
$$u_2(y) = \frac{K_1\left(\gamma - (k_1 + k_2)\right)}{2k_2} e^{(\gamma - k_1 - k_2)y/2}\,. \tag{7}$$

<u>Intermediate region ($K_1 < u < K_2$).</u> In this region $f(u) = 0$ and $g(u) = 1$, leading to

$$\frac{\mathrm{d}u}{\mathrm{d}y} = k_2 u_2\,,$$
$$\frac{\mathrm{d}u_2}{\mathrm{d}y} = -k_2 u_2\,.$$

Hence, $u_2(y) = Ce^{-k_2 y}$ for a constant C, which can be found by continuity at $z = 0$. We obtain

$$u_2(y) = \frac{K_1\left(\gamma - (k_1 + k_2)\right)}{2k_2} e^{-k_2 y}\,. \tag{8}$$

Given that this is a decreasing exponential, we have found that the peak in S/G2/M cell density, ρ_2^{edge}, occurs at $y = 0$. This is, $\rho_2^{\mathrm{edge}} = u_2(0)$.

Since in this region $u + u_2$ is a constant, which can be found by continuity at $y = 0$, we also obtain

$$u(y) = K_1 + u_2(0) - u_2(y)\,.$$

<u>Tissue bulk ($K_1 < K_2 < u$).</u> Now we have $f(u) = g(u) = 0$ and hence we can write

$$\frac{\mathrm{d}u}{\mathrm{d}y} = \frac{\mathrm{d}u_2}{\mathrm{d}y} = 0\,.$$

In the tissue bulk, both densities are constant and $u = K_2$. By using continuity, and the solutions from the intermediate region, we find

$$\rho_2^{\mathrm{bulk}} := u_2(z) = \left(K_1 - K_2 + u_2(0)\right)_+\,, \tag{9}$$

where we impose positivity of $u_2(z)$.

In particular, we find that, whenever $\rho_2^{\mathrm{bulk}} > 0$, the S/G2/M cell density difference

between the tissue edge and the bulk satisfies

$$\rho_2^{\text{edge}} - \rho_2^{\text{bulk}} = K_2 - K_1 \,.$$

In this case, and by combining Eqs. (7), (8), and (9), we obtain the full solution

$$u_2(y) = \begin{cases} \frac{K_1(\gamma-(k_1+k_2))}{2k_2} e^{(\gamma-k_1-k_2)y/2} \,, & y \leq 0\,; \\ \frac{K_1(\gamma-(k_1+k_2))}{2k_2} e^{-k_2 y} \,, & 0 < y \leq y^*\,; \\ \frac{K_1(\gamma-(k_1+k_2))}{2k_2} - (K_2 - K_1)\,, & y > y^*\,, \end{cases} \tag{10}$$

for $y = -(x - ct)/c$, and y^* defined from Eq. (8): $k_2 y^* = \log(\rho_2^{\text{edge}}/\rho_2^{\text{bulk}})$.

S5 Supplementary Figures

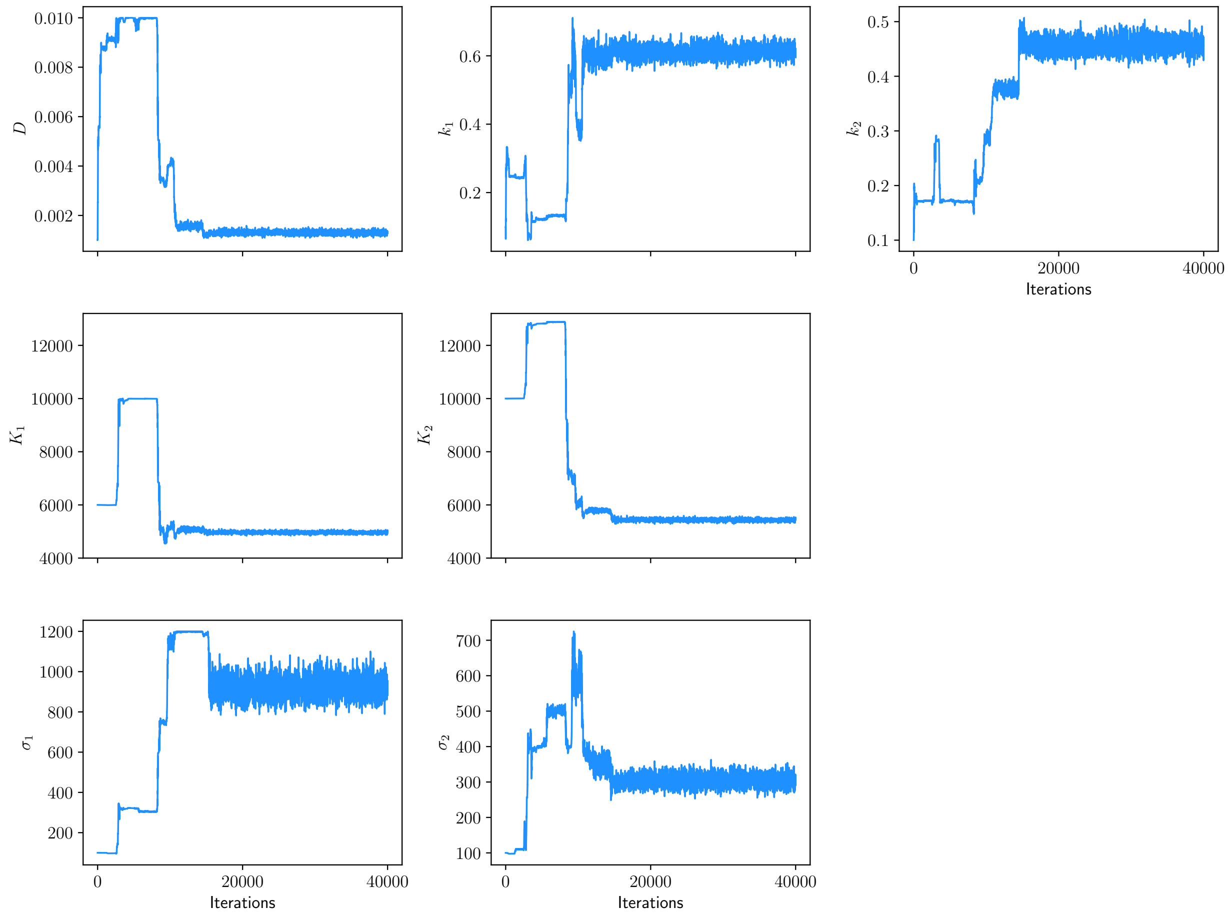

Figure S1: MCMC iterations for the large tissue expansions experimental data. Parameters D, k_1, k_2, K_1, K_2 correspond to the model presented in the main text, and σ_1, σ_2 are error model parameters.

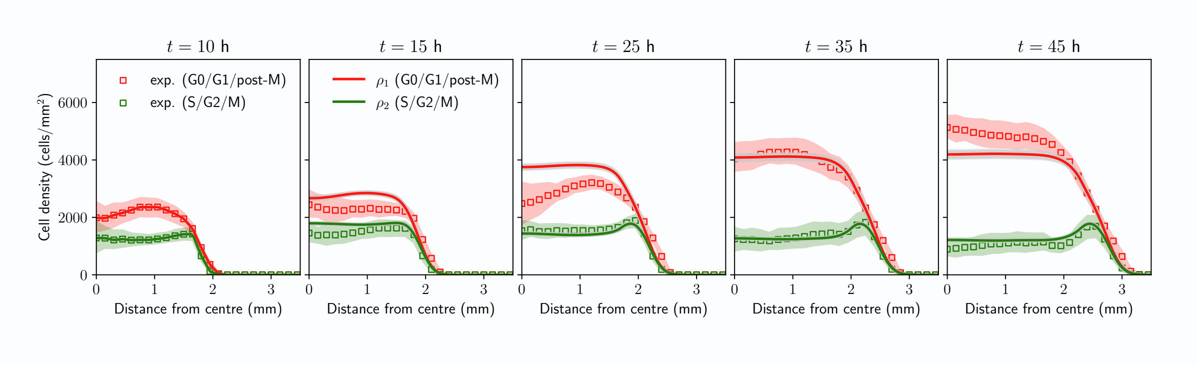

Figure S2: Comparing data (squares) and model predictions (solid lines) for large tissue expansions. Coloured shaded regions denote one experimental standard deviation with respect to the mean, obtained by averaging eleven experimental realisations. Gray shaded regions represent 95% confidence intervals obtained from the posterior distributions.

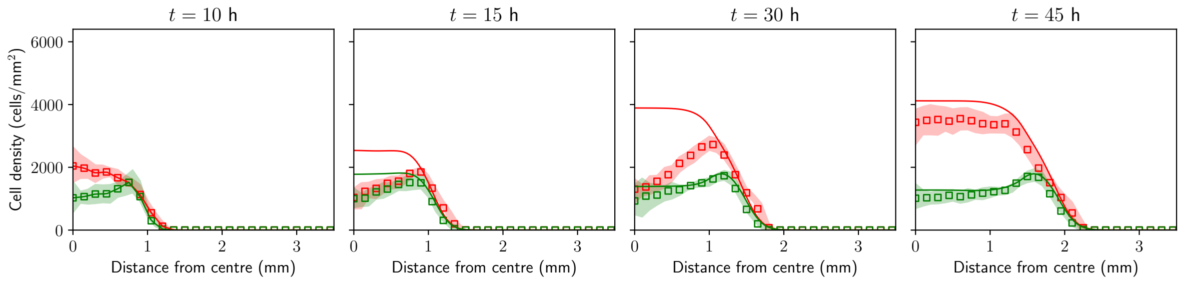

Figure S3: Comparing data (squares) and model predictions (solid lines) for small tissue expansions. Shaded regions denote one standard deviation with respect to the mean, obtained by averaging five experimental realisations. Numerical simulations in polar coordinates were obtained by using the parameter values obtained from the large tissue expansions, and no-flux boundary conditions. In order to minimise the effects of the stencil removal on cell behaviour, the initial condition corresponds to the experimental density profile ten hours after stencil removal.

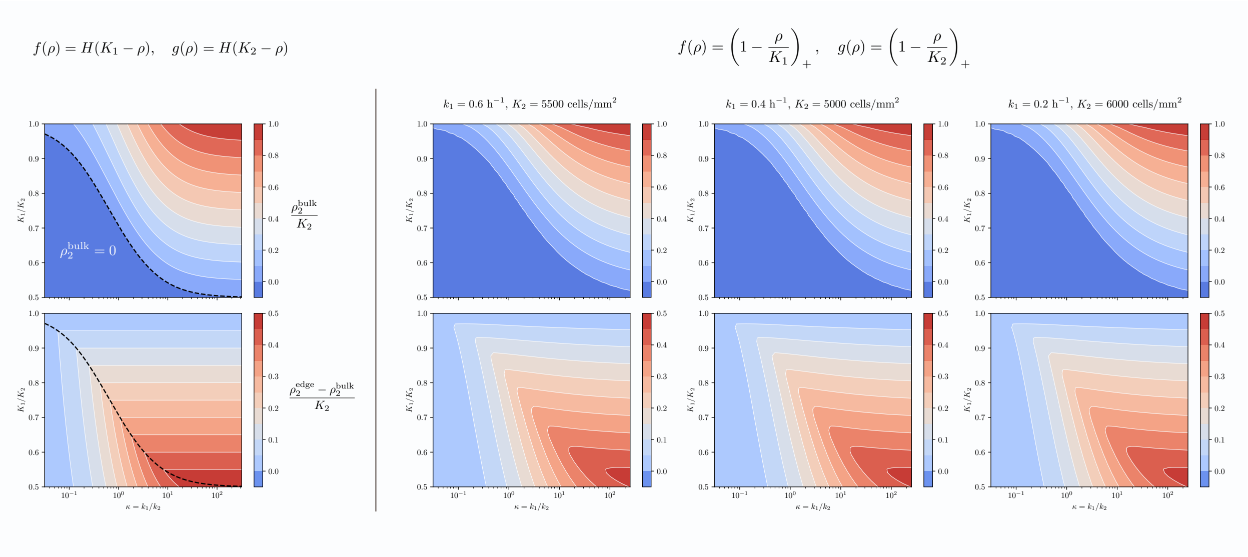

Figure S4: S/G2/M cell densities at the tissue edge and tissue bulk as a function of the ratios $\kappa = k_1/k_2$ and K_1/K_2: comparison between the simplified model (left) and the model presented in the main text (right). For the model with $f(\rho) = (1 - \rho/K_1)_+$, $g(\rho) = (1 - \rho/K_2)_+$, plotted values are obtained by solving numerically Eqs. (5) and (6) with different parameter values, as indicated in the figure. These results confirm that ρ_2^{edge} and ρ_2^{bulk} are determined by the two ratios of parameters: k_1/k_2 and K_1/K_2.

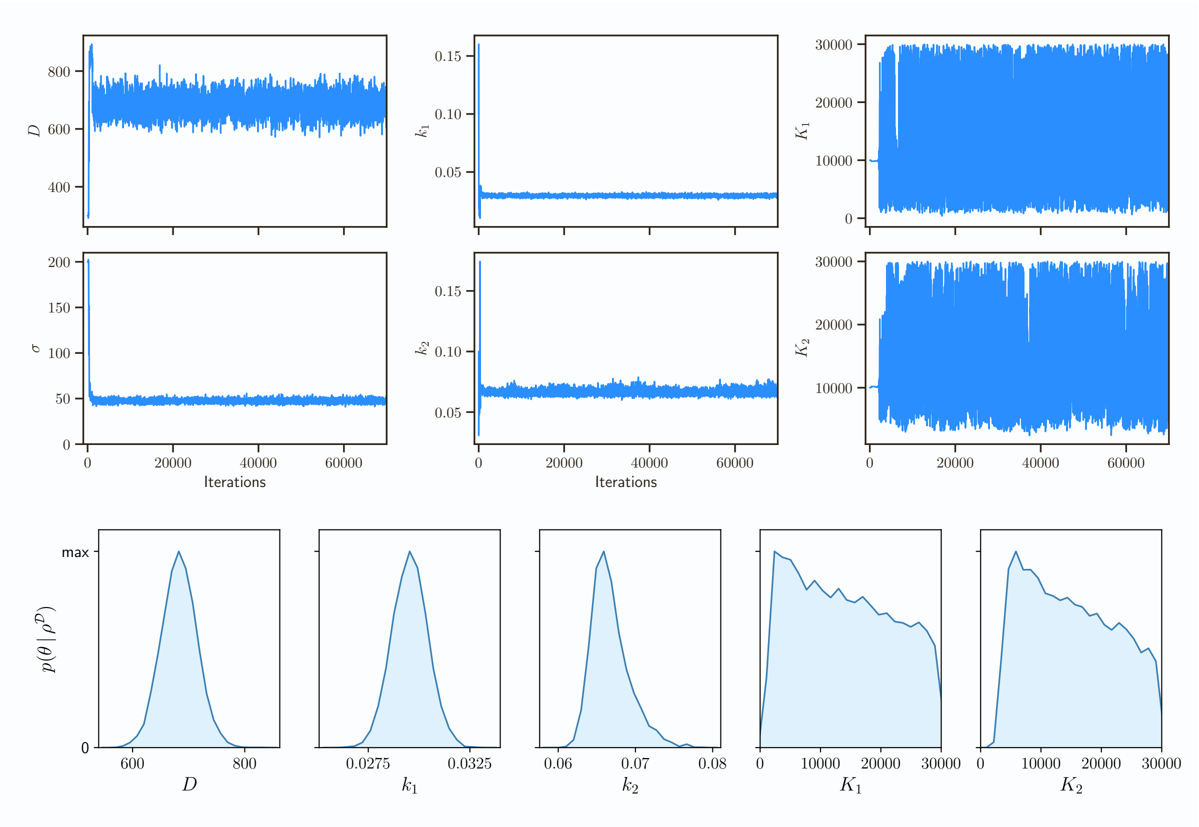

Figure S5: MCMC iterations and univariate marginal posterior distributions obtained by using low-density scratch assay data [8]. With this dataset, K_1 and K_2 are practically non-identifiable.

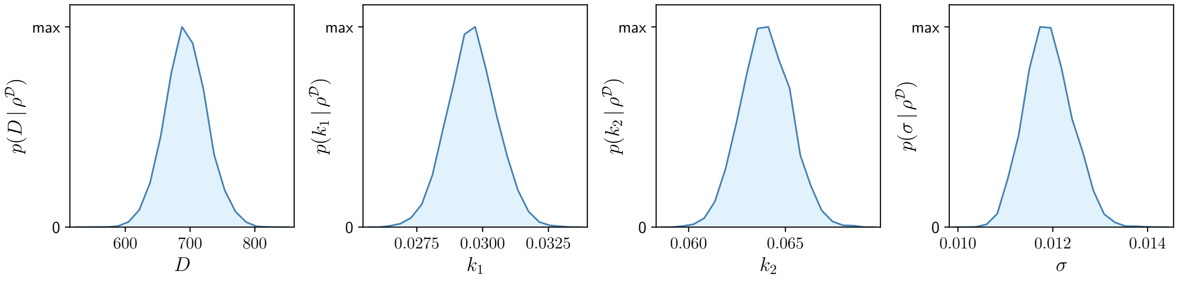

Figure S6: Univariate marginal posterior distributions for the exponential growth model ($f(\rho) = g(\rho) = 1$) obtained by using low-density scratch assay data [8]. In this case, the model has three parameters (D, k_1, k_2) and one error model parameter (σ, normalised by the theoretical maximum density assuming hexagonal packing of cells [10]).

References

[1] J. Canosa. On a nonlinear diffusion equation describing population growth. *IBM Journal of Research and Development*, 17(4):307–313, 1973.

[2] M. A. Heinrich, R. Alert, J. M. LaChance, T. J. Zajdel, A. Košmrlj, and D. J. Cohen. Size-dependent patterns of cell proliferation and migration in freely-expanding epithelia. *eLife*, 9:e58945, 2020.

[3] B. Lambert, C. L. Lei, M. Robinson, M. Clerx, R. Creswell, S. Ghosh, S. Tavener, and D. J. Gavaghan. Autocorrelated measurement processes and inference for ordinary differential equation models of biological systems. *Journal of the Royal Society Interface*, 20(199):20220725, 2023.

[4] J. D. Murray. *Mathematical Biology I: An Introduction.* Springer New York, 2001.

[5] S. Schönfeld, A. Ozkan, L. Scarabosio, M. N. Rylander, and C. Kuttler. Environmental stress level to model tumor cell growth and survival. *Mathematical Biosciences and Engineering*, 19(6):5509–5545, 2022.

[6] S. Schönfeld, L. Scarabosio, A. Ozkan, M. N. Rylander, and C. Kuttler. Using systemic modeling and Bayesian calibration to investigate the role of the tumor microenvironment on chemoresistance. *arXiv preprint arXiv:2310.19688*, 2023.

[7] Y. Schälte, F. Fröhlich, P. J. Jost, J. Vanhoefer, D. Pathirana, P. Stapor, P. Lakrisenko, D. Wang, E. Raimúndez, S. Merkt, L. Schmiester, P. Städter, S. Grein, E. Dudkin, D. Doresic, D. Weindl, and J. Hasenauer. pyPESTO: a modular and scalable tool for parameter estimation for dynamic models. *Bioinformatics*, 39(11):btad711, 2023.

[8] M. J. Simpson, R. E. Baker, S. T. Vittadello, and O. J. Maclaren. Practical parameter identifiability for spatio-temporal models of cell invasion. *Journal of the Royal Society Interface*, 17(164):20200055, 2020.

[9] M. J. Simpson, R. J. Murphy, and O. J. Maclaren. Modelling count data with partial differential equation models in biology. *bioRxiv 2023.09.09.556963*, 2023.

[10] S. T. Vittadello, S. W. McCue, G. Gunasingh, N. K. Haass, and M. J. Simpson. Mathematical models for cell migration with real-time cell cycle dynamics. *Biophysical Journal*, 114(5):1241–1253, 2018.
